# Researcher identities and values in the impact agenda: the case of artificial intelligence academics

**DOI:** 10.1007/s10734-024-01356-1

**Published:** 2024-11-12

**Authors:** Eliel Cohen, Kate Williams, Jonathan Grant

**Affiliations:** 1 The Policy Institute, King’s College London, London, UK; 2 School of Social and Political Sciences, University of Melbourne, Melbourne, Australia; 3 Different Angles Ltd., Cambridge, UK

**Keywords:** Research impact, Academic identity, Policy, Artificial intelligence

## Abstract

A major shift in the research sector has been the increased expectation from policymakers and funders that academic research should yield some socioeconomic benefits or ‘impacts’ rather than merely new knowledge. In this paper, we explore the role that impact has in academics’ motivations and values and how impact is being integrated into academics’ core functions of research and education. We do this through in-depth interviews (*n* = 60) with scientists who work on the development or application of artificial intelligence (AI), broadly defined. This AI’s focus situates our participants within a strategically important, high-priority area of research for all three national contexts included in our study—Australia, the UK and the USA. Our findings reveal that the impact mission has become central to understanding the motivations and values of academics, but unevenly. We identify divergence between those who work on AI from a foundational computer science perspective and those who develop and apply AI within other scientific domains. The two groups have different understandings of key notions such as ‘impact’ and ‘applied research’, as well as different ways of integrating the impact agenda into their research and education activities. The study highlights the importance of flexible approaches to research policy and governance that are based on a deeper understanding of what motivates researchers, and that take into account academics’ educational role. Greater holistic understanding of how academic identities and practices are accommodating the impact agenda is essential to maximise synergy across activities and avoid unintended consequences.

## Introduction

A major shift that has affected the university sectors in recent decades has been the increased expectation that publicly funded research should yield demonstrable socioeconomic benefits or ‘impacts’ rather than merely new knowledge. While our empirical focus is Australia, the United Kingdom (UK) and United States of America (USA), a number of systems have also adopted very similar impact policy discourses and initiatives that can be understood as part of a broader ‘impact agenda’ (most explicitly in Hong Kong, Italy, Poland and Norway, but the tendency is far broader). These policies align with theories of a global tendency towards closer ties between research and applied contexts and a weakening of the traditional barriers between science and society, most notably the ‘mode 2’ thesis (Bentley, Gulbrandsen & Kyvik, 2015). We discuss our findings with reference to this mode 2 thesis, which we introduce below. However, our main interest is whether and how national research policies with this objective, i.e. the impact agenda, interact with and potentially influence aspects of academics’ sense of their values and identities. In doing so, we contribute to ongoing work exploring the link between strategic policies affecting academia on the one hand, and academic identities on the other (e.g. Balaban & de Jong, 2023; Ben-David, 1971; Chubb et al., 2017; Watermeyer, 2019). Our main theoretical reference point in the higher education literature is Henkel (2000a, b, 2005) who analysed ‘the implications of [research] policy changes for academics, their values, agendas and self-perceptions, in other words for their academic identities’ (200a, p. 9). In our discussion, we take this a step further by suggesting that we should not only focus on how such policies may affect identities, but also consider what implications academic identities have for the intended outcomes of policy objectives.

We focus on a group of academics whose research situates them not only in an area of significant potential impact, but at the intersection of several national policy initiatives, namely scientists who work on artificial intelligence (AI), broadly defined. AI is a strategically important, high-priority area of research for all three national contexts included in our study. AI is also inherently multidisciplinary, which is another strategic focus for all three countries, and relates to multiple areas of social and economic life. This relevance to multiple science policies puts this group in a position of privilege, but also exposes their research to political interventions. Before describing our study, we discuss the most relevant recent policy trends and how existing literature shapes our current understanding of the relationship between research policies and academic identities and values.

### Key drivers and trends towards the impact agenda in national policies for academic research

The main proclaimed drivers for academic research policies in recent decades have been economic growth and enhanced public accountability. For example, on the economic side, the USA’s 1980 Bayh-Dole Act attempted to stimulate economic activity and academic entrepreneurship by permitting private ownership of inventions arising from government-funded research. By the late 2000s, countries across all populated continents had put into place similar arrangements. On the public accountability side, the year 1993 has been highlighted as a turning point in both the USA, with its Government Performance and Results Act (Watts et al., 2015) and the UK, with its ‘Realising our Potential’ White Paper (Hill, 2016). Both nations’ research funding agencies—the USA’s National Science Foundation (NSF) and Research Councils UK (now UK Research & Innovation [UKRI])—responded to State drivers by instituting non-academic ‘impact’ as part of their funding criteria (Hill, 2016; Watts et al., 2015). More recently, the UK’s Research Excellence Framework (REF) and Australia’s Excellence in Research for Australia (ERA) have led on instituting systematic accountability and incentive structures via national-level ex post assessments of non-academic impact (Williams & Grant, 2018). This involves assessing and ranking departments and universities based on non-academic impact as well as on academic accomplishments.

Not all drivers are economic, nor are they all top-down, and academics have not only received but have also contributed to the creation of demand for more impact-oriented research, for example by seeking revenue-generating activities and producing doctoral graduates who enter the private sector (Gibbons, 2000). However, a focus on the relationship between research policy and researcher identities and values is important because national policies designed to steer academic research activity cannot be successful without, to some extent, acting upon the nature of academia itself, given that autonomy is central to academia’s self-understanding. As Henkel (2005) put it, the notion of academia as a ‘self-regulating’ community became unsustainable ‘as higher education and science became increasingly important instruments of national economic policy’ (p. 159). From this perspective, the policy challenge is how to engender cultural shifts that help harness socioeconomic benefits of academic knowledge without undermining what is distinctively valuable about academia and what made academic research such an important area for national policy interventions in the first place.

### The relationship between research policy and research activities through the lens of academic identity and values

This focus on links between science policy and academic identity emerged primarily in the UK and European context (e.g. Henkel, 2000b; Kogan, Bauer, Bleiklie & Henkel, 2000), although it was part of a broader conversation with authors covering similar developments in different national contexts (e.g. Etzkowitz & Leydesdorff, 1997; Marginson & Considine, 2000; Slaughter & Leslie, 1997). Henkel’s (2000a, 2005) theoretical framing is of most relevance to this paper. Drawing on communitarian philosophies of identity (e.g. MacIntrye, 1981; Taylor, 1989), she focused on what provides academics with their sense of identity and meaning, what they see as the main purpose and value of their research, and who their most relevant community/ies and audiences are. For example, she argued that national science policies starting from around the 1970s and 1980s had contributed to an increased complexity and decreased stability of academic identities, i.e. a weakened attachment to formalised institutional structures such as the university and the department, replaced by more nebulous attachments, such as discrete projects involving different collaborators and stakeholders. However, she also stressed important continuities with the past, arguing that the discipline ‘remains a strong source of academic identity, in terms of what is important and what gives meaning’, and that ‘major changes in the funding of research and the context in which it is carried out have not created major disturbances in academic values or academic identities’ (Henkel, 2005, p. 174).

Henkel’s (2005) conclusions come from her interviews with 54 UK-based biologists and biochemists. She reported that all but one of these academics framed their prime research motivation in terms of advancement of basic knowledge, with impact goals such as clinical and commercial outcomes being secondary or marginal objectives. Such findings seem counter to the widely cited Mode 2 thesis (Gibbons et al., 1994; Nowotny et al., 2001), which emphasises significant shifts in knowledge production and science-society relations. Gibbons (2000) summarises the thesis as follows:

‘in Mode 1, problems are set and solved in a context governed by the, largely academic, interests of a specific community. By contrast, in Mode 2, knowledge is produced in a context of application involving a much broader range of perspectives; Mode 2 is transdisciplinary, not only drawing on disciplinary contributions but can set up new frameworks beyond them… It is more socially accountable and reflexive than Mode 1. …Mode 2 includes a wider, more temporary and heterogeneous set of practitioners, collaborating on a problem defined in a specific and localised context.’ (pp. 159-160)

Indeed, around a decade after Henkel’s study, a survey including 7000 UK STEM academics found that the most common description that participants selected for their research was ‘applied’, with 35% selecting this option, as well as 34% selecting the term ‘user-inspired basic’ and only 31% selecting the term ‘basic’ (Hughes & Kitson, 2012).^1^ This would suggest that significant changes had occurred in that decade in ways that support the mode 2 thesis. However, it is difficult to infer from such results whether they represent a fundamental change in research values and objectives or a change in accepted terminology. Indeed, one of Henkel’s (2005) arguments is that academics are willing to frame aspects of their research in policy terms, not because of a sudden shift in values but to gain opportunities for professional autonomy, such as independent research funding. Likewise, Lam (2011) argues that academics have often been able to capitalise on external communities’ interests and investments to satisfy their ‘psychological need for autonomy’ (p. 1356). In such theories, ‘impact’ as a criterion of success becomes a new form of scientific or academic ‘capital’ (Papatsiba & Cohen, 2020; Williams, 2020).

Qualitative studies conducted after the introduction of ‘impact’ into national policy discourse and evaluation (e.g. of Australia and UK) reveal that many academics merely comply with the impact mission (Chubb & Watermeyer, 2017; Leathwood & Read, 2013; Oancea et al., 2017). The studies emphasise the ‘performative’ nature of compliance. Strategic compliance, where impact is a means of advancing research interests and academic autonomy, has given way to performative compliance, whereby the impact agenda occurs at a more surface level. This suggests that while some academics see impact policies as meaningfully aligned with their research and career goals, others—perhaps the majority—simply adhere to the new expectations. Cohen (2021, 2024) also highlights the emergence of a group for whom impact is central, and who define their identities in opposition to the lack of interest and incentives for impact that they perceive as characterising academia.

When attempting to understand the relationship between research policies and researchers’ values, it is difficult to disentangle genuinely held values from the ways that academics articulate and position their goals and values in relation to policy. Indeed, there may be systematic differences between groups that do not fall neatly along typical lines (e.g. hard/soft disciplines, applied/basic). Henkel (2000a) notes that shifts at the level of research policies and categories (e.g. the proportion of research characterised as ‘applied’ vs. ‘basic’) do not necessarily imply equally significant shifts in academics’ sense of identities or values. Thus, ongoing research into this topic is required to understand academics’ adaptation to changing socioeconomic and policy environments and to understand the effects and effectiveness of national policies.

## Study design and methods

### Sampling

This paper reports on a subset of interviews as part of a wider study of 90 academics—30 from each Australia, the UK and the USA—spanning a range of so-called ‘STEMM’ (science, technology, engineering, mathematics and medicine) disciplines and social sciences and humanities. All participants were selected based on having a shared specialist focus on some aspect of the development, application or implications of AI. In this paper, we focus on 60 STEMM researchers. This is because we found notable variations within the STEMM group that merited its own analysis and interpretation. While there is value in analysing a broader range of disciplines (e.g. Chubb & Watermeyer, 2017), comparative studies with fewer disciplines allow for the identification and articulation of more nuanced forms and sources of variation (e.g. Balaban & de Jong, 2023; Cohen, 2021; Oancea et al., 2018). Future work will report on other aspects of the wider study, foregrounding cross-disciplinary and cross-national features.

In each country, we identified two institutional settings that exemplified the high-priority nature of AI: one multi-university network in receipt of strategic national funding; and one university with a highly visible and well-funded internal multi-departmental AI network (see Table 1).

**Table 1:** Selection of institutional settings

	National AI/automation networks	University networks with AI focus
Australia	ARC Centre of Excellence in Automated Decision Making and Society	University of Queensland (UQ)
UK	Alan Turing Institute	University College London (UCL)
USA	NSF AI Institutes	New York University (NYU)

The three multi-university networks are the NSF AI Institutes, which include several hundred affiliated researchers; the Alan Turing Institute, with around 600 affiliated researchers; and the Centre for Automated Decision Making and Society, with nearly 100 affiliated researchers.^2^ The three multi-departmental networks are NYU’s ‘AI Initiative’, a virtual network with strategic input from university leadership, with around 100 affiliated staff across nine departments; UCL’s ‘AI for People and Planet’ network, with 80 affiliated researchers; and the ‘AI at UQ’ network, with 114 academics spanning twenty departments. Table 2 presents the participants by national context and by ‘computer science’ and ‘other STEMM’ backgrounds. The USA is the most represented national context followed by the UK and Australia. The ‘other STEMM’ category (i.e., non-computer science) includes academics from physics, biology, chemistry, cognitive and computational psychology, clinical, environmental and agricultural sciences and engineering fields.

**Table 2:** National and disciplinary context of participants

National context	Computer science	Other STEMM	Total (% of sample)
Australia	7	7	14 (23%)
UK	11	9	20 (33%)
USA	17	9	26 (43%)
**Total (% of sample)**	**35 (58%)**	**25 (42%)**	**60 (100%)**

We focused mainly on academics who were well established in their careers, in order to access experiences of engaging with and reflecting upon the impact agenda and its context, i.e. how policy, expectations or culture has shifted over time. The 60 participants include 57% professors, 12% at reader or associate professor level, 23% at lecturer or assistant professor level and 8% research fellows.

### Recruitment and ethics

Invitations to participate and an information sheet were sent via email. Interviews were conducted in person in the participant’s workplace or via video call. Consent was gained, and all interviews were audio recorded and transcribed, except for one who preferred that only notes of the interview be taken. Ethical clearance was provided by the corresponding author’s Research Ethics Committee (reference number MRA-20/21–23,367).

The key ethical consideration in this study is anonymity. We limit information about the participants and include only reference to broad disciplinary area (computer science or ‘STEMM’) and the national context in which they work.

We also feel an ethical responsibility that our conclusions are representative of all participants. In particular, our 60 participants included only 12 women. We stress that the claims made in this paper apply to the women in our study as well as the men—we do not note any systematic difference by gender on the themes raised in our findings.

### Interviews

The interview protocol was designed to allow our participants to express their values in relation to their research and academia in an authentic way. Our opening e-mail and information sheet explained that we had an interest in research policies and evaluation criteria, specifically around impact. However, we did not open our interviews with questions around impact, instead asking broader open questions. Interviews were semi-structured so did vary from interview to interview but most followed the following protocol. First, we asked participants questions that invited them to summarise their research trajectory, current main research topics and aims, and what their typical research activities practically involve, i.e. key methods, collaborations, outputs, etc. Second, we asked more reflective questions about general research motivations and what our participants consider to be the key values associated with their research, as well as what might count as an indicator of the value of research. Third, we asked about which aspects of their working environment and culture enable or potentially hinder their ability to work towards what they consider ‘valuable’ outcomes. Some more details about how specific questions were introduced are provided where relevant in the Findings section. This was trialled in two pilot interviews, which achieved a balance between explaining the context of our study whilst also allowing participants to discuss their values and motivations on their own terms. In most interviews, the above questions led to participants raising the issue of the non-academic impact of their work, and policies related to this, but where it did not, we asked participants directly about their views on such policies.

The findings are mainly based on the responses we received to our questions about participants’ motivations and what they personally find to be the most valuable aspects of their research. Interviews lasted approximately 1 h although occasionally closer to 30 min or 2 h.

### Analytical approach

Analytically, the objective of interviews was to tease out distinctions between:

I. The main motivations driving participants’ research
II. The key values that they have in relation to their research and the academic profession
  A. particularly those values that they see as most central to being an academic
  B. or as being potentially under threat
III. The key indicators of the value of research—outcomes that make them feel that they have done valuable or meaningful work, whether formal metrics or informal/personal indicators.

We inductively coded instances that could relate to the above three areas and iteratively grouped similar motivations and values together to distil commonalities and divergences. In some areas, there were keywords that were frequently used to express motivations and values. Elsewhere, identifying similar meanings required careful interpretation, and in such cases, we present and contextualise quotations.

We also inductively coded participants’ perspectives on the issue of impact, separating out different elements: impact as a motivation, goal or (indicator) of value; impact as a policy and criterion of assessment; impact as an academic activity potentially complementary to other key academic activities (e.g. the production of new knowledge and education). As such, our approach was informed by theory of the link between academic research policy and researcher identities and values as well as emerging insights from the data.

## Findings

We organise our findings around three themes. First, we explore the role of ‘impact’ in descriptions of research motivations and desired outcomes. Our open questions about participants’ values and motivations provide insight into the significance (or otherwise) of impact-related issues. Second, we explore the link between impact and knowledge advancement activities. This gives insight into whether impact is an add-on activity or more deeply integrated into research practices and purposes. Third, we focus on impact as it relates to education. The education function of universities emerged as a major theme in our interviews, allowing exploration of how the impact agenda is being integrated with academics’ identity as educators.

We also organise the findings along a disciplinary dimension. This is informed by the above theoretical literature which frames disciplinarity as a key traditional source of academic identity, community and values, but one which has been under pressure in the context of recent national policies in all three selected countries. However, it is also informed by the data, since one of our most striking findings is the consistent divergences between our participants from computer science backgrounds on the one hand, and other STEMM disciplines on the other.

For the themes discussed in this paper, these disciplinary variations are far more apparent than national differences. Indeed, we were struck by the similarity across national contexts and plan future work analysing this individual-level uniformity given system-level differences. Moreover, qualitatively, participants described their motivation more in relation to the epistemic/disciplinary content of their work than the national policy context. Similarly, although there are some differences between the 57% of respondents who are full professors and the 43% who are not, these do not play out significantly in the themes discussed in this paper. Generally, more senior academics from both the computer science and other STEMM researchers are more likely to mention a broader range of goals, motivations and values than less senior researchers, but the relative differences between computer scientists and other STEMM researchers holds across career levels.

### Impact as a motivation and indicator of value

#### ‘Impact-oriented’ vs. ‘intellectual’ research motivations and research values

Much of the empirical literature reviewed above involved attempts to understand academics’ motivations and values towards impact in relation to their values towards other objectives such as contributing to a discipline, theory or knowledge for its own sake. To contribute to this body of knowledge, we group together all our interview participants’ responses that fall under each type of motivation and illustrate this with both descriptive statistics and direct quotations.

Table 3 summarises the percentages of the most frequently mentioned categories: ‘impact-oriented’ and ‘intellectual’ research motivations and values. It shows the percentages for both groups, computer scientists and other STEMM participants. Since many participants expressed both impact-oriented and intellectual motivations and values, the figures in parentheses refer to the percentages of participants who expressed one type as a clear priority. The table shows that computer scientists emphasise intellectual over impact-oriented (86% vs. 71%), while other STEMM participants in our study overwhelmingly emphasise the reverse (68% vs. 92%).

**Table 3:** Participants’ mentioning impact and curiosity as a main and as their primary (in parentheses) motivation for research

Research motivation/value	Computer scientists	Other STEMM researchers
Impact-oriented	71% (40%)	92% (56%)
Intellectual	86% (57%)	68% (28%)

The below quotations illustrate computer scientists’ intellectual research motivations and values. They describe being motivated by ‘curiosity’ and the thrill of problem-solving and emphasise their personal, even idiosyncratic, interests in pursuing particular topics and research avenues.

‘I choose to do the research that I think is most interesting’ (Participant 49, CS, UK)

‘The ability to solve a problem that no one has ever solved before’ (P69, CS, Australia)

‘I personally like to tackle complicated things, so the intellectual excitement is absolutely a dopamine part of the job’ (P34, CS, UK)

Indeed, 54% of computer scientists mentioned ‘freedom’ or ‘autonomy’ as an important value for them as academics and researchers, compared to only 24% of STEMM participants.

In addition to personal autonomy and intellectual stimulation, computer scientists also emphasise being motivated by intellectual contributions. This highlights that computer scientists think about the value of their research in terms of their immediate discipline:

‘You see someone *using* your paper, not just citing it… we’re having decent intellectual conversation’ (P14, CS, USA)

‘I want to make the discipline as good as it can be, like, … how can we change the processes we use and so on to improve?’ (P49)

‘The question [of how worthwhile my research is] is, to what extent have I done something that’s been useful or interesting to the [research] community’ (P62, CS, USA)

More than one-third (36%) of the computer scientists related this disciplinary focus to the view that ‘quality’ of contribution and engagement was more important than quantitative metrics such as the number of outputs or citations. By comparison, only 12% of STEMM participants made a similar point.

#### Qualitative divergences in the role of impact-oriented research motivations and values

Beyond differences in the frequency with which computer scientists and STEMM mention impact-oriented motivations and values, the meaning that the two groups attached to impact also diverge. STEMM participants are frequently direct and explicit, often using the words ‘impact’ and ‘application’. Here, specific areas or types of impact and application play a primary role in orienting their research and their evaluation of it:

‘I do highly value the impact of the research. And especially as I’m doing applied research, I will want to see what actual value is my original model bringing.’ (P70, STEMM, UK)

‘So for me, a job well done is something that has a high chance of integrating into some workflow that can change people’s lives’ (P29, STEMM, UK)

‘for me, the key driver that actually motivates us to do the research is we identify problems or issues that the … industry has and we try to tackle those issues’ (P21, STEMM, Australia)

By contrast, computer scientists are less direct and more generic (albeit not necessarily less passionate, as later sections show) about their potential broader impacts. Computer scientists seem less motivated by specific intended outcomes or impacts. While other STEMM participants often centre specific user/beneficiary groups as key communities or publics, computer scientists describe their potential beneficiaries in more generic terms, such as wanting to ‘improve lives’ or ‘address problems’:

‘…how are we going to use computer science to improve lives? And that could mean a number of different things’ (P38, STEMM, USA)

‘I think that there are lots of problems that we should be solving and that it's unethical for us to be wasting brainpower and computer power and students’ time on something that is just for the purpose of publishing. So I think we have that responsibility really to be addressing problems’ (P53, STEMM, USA)

### The integration of the impact agenda in research

#### Impact as deeply embedded in STEMM participants: a ‘localising’ approach to integrating impact and research

Since one of the goals of impact policy is to encourage impact outcomes to become more integrated into research culture, practices and aims, we explore the ways in which impact features in our participants’ accounts of their research. Here, we also find important divergence between STEMM and computer scientists.

STEMM participants had quite significantly integrated the impact agenda into their research practice and outcomes. At an aggregate level, participants differed in how they classified their research; 76% of STEMM participants described their work as ‘applied’, compared with only 54% of computer scientists. Moreover, ‘application’ appears not just to be a descriptive classification but a core principle of how research agendas get set and how it gets done.

One widely shared feature of STEMM participants’ research was orientation towards problems and questions derived from contexts of application or practice. For example, two had deliberately sought out roles situated in health-related research departments despite having non-health backgrounds because they wanted direct access to practitioners—or ‘the problem holders’ (P29), as one participant called them—to better orient their research to clinical needs.

The impact mission can provide a new principle for aspects of research such as what counts as legitimate collaborations and dissemination channels. For example, one explained that although they had issues ‘in principle’ with working with private for-profit companies, they nevertheless did so because it ‘can be a faster pathway to impact’ (P82, STEMM, Australia).

P29 described how localised knowledge of the context of application is obtained by close ‘direct interaction’ within the user’s physical workplace, allowing them (P29) to ‘keep developing solutions’. For P29, this ‘is why we [me and my team] embedded ourselves in academia. To understand the fundamentals and then use that fundamental understanding to basically develop practical solutions.’

Indeed, in the above case, the industry’s demand for scientific knowledge not only shapes the participant’s research agenda, but also underpins their motivation for choosing to work in a university. This suggests that the impact agenda is not only leading to changes in the trajectory of academics’ research and careers but also influencing the kinds of people who enter the academic profession.

For the STEMM cases above, their integration of impact into research involved career-long engagement with contexts of practice and application, as well as deep and ongoing interpersonal connections with professionals at these sites. We label this STEMM approach a ‘localising’ approach, in contrast with the ‘generalising’ approach to integration amongst computer scientists.

#### Impact as linearly related to research by computer scientists: a ‘generalising’ approach

For the STEMM participants, the work of applying knowledge and attempting to generate impact was often central to their core activities and hard to disentangle from ‘research’ or ‘knowledge-advancement’. By contrast, computer scientists had a very different notion of what counts as ‘applied’ research. For them, ‘applied’ typically simply meant that the research involved ‘working with datasets’ (P60, CS, USA) as opposed to being purely theoretical. There was a sense of a clear division between theoretical and empirical, with the latter often being taken to imply or assume ‘applied’; this point was explicitly made by several computer scientists, as was the sense of division between ‘theoretical’ and ‘empirical’, where research stops being ‘theoretical’ once it involves data and real-world contexts; for example, computer scientists used phrases such as ‘I’m not much of a theoretician, I work more on the empirical, the more applied’ (P37), ‘we’re interested in … establishing theoretical properties where we can, but the majority of my work is more empirical … driven by applications’ (P2).

This notion of application and impact as being linearly ‘downstream’ from theoretical research is largely absent in most STEMM participants, but was common amongst computer scientists, two of whom explicitly used this term to describe their understanding of impact (P35, P37) and indicates a very different approach to thinking about and integrating the impact mission into research. For computer scientists, the label ‘applied’ is not defined by its actual proximity to or likelihood to influence contexts of application, but simply the application of theories or algorithms to data. Several also described collaborations with researchers from other STEMM discipline as being ‘applied’, even if the research objectives were towards fundamental understanding rather than application. One participant even acknowledged this fact openly, reflecting that such collaborative research is often ‘blue skies’ but that they call it ‘applied’ in order to ‘put a narrative on how one thing follows from the other’.

Some computer scientists aim to more deeply connect their research to contexts of application. However, this takes a very different form to STEMM. By contrast to the localising approach of STEMM participants, computer scientists exhibited a ‘generalising’ approach, whereby impact is integrated into research to the extent that researchers aim to create algorithms that can have broad potential relevance across a number of areas, rather than a researcher requiring deep knowledge of one or two particular domains. One computer scientist described themselves as being ‘driven by the application I’m working on [at a given time]’, applying generalisable algorithms across a ‘variety of areas, that goes from a clinical situation, education, fashion, sustainable industry, games and many more’ (P52, CS, USA). As another participant put it, ‘the best results in AI and machine learning are results that are generalizable, so a technique that can be applied to problems in lots of different areas’ (P47, CS, Australia).

Another way of conceiving this ‘generalising’ approach of computer scientists could be a linear ‘theory-driven’ approach to application and impact, where impact and application are largely distinct from and linearly downstream of research. This is not to say that the applications and impacts of our STEMM participants are not underpinned by theory or do not involve theoretical work. Rather, the point is that our STEMM participants do not describe impact/application as being a linear application of theory, but rather as about seeking scientifically challenging research problems within contexts of application and in collaboration with ‘problem-holders’ who work in these contexts.

### Impact in relation to education

The educational function of universities and how this shapes academics’ values and motivations, including those around research, was a major theme in the interviews. Almost all participants mentioned valuing and being motivated by their educational role, with the most common themes being a strong research-teaching relationship and the importance of this relationship for the realisation of research impact. Our analysis suggests that how computer scientists and other STEMM participants talk about education and its relation to impact aligns with each group’s broader values and motivations as discussed above.

#### STEMM integration of the impact mission and education function

In line with their strong impact-oriented motivations and values, our STEMM participants often spoke of students and graduates in terms of mechanisms for impact. The clearest examples mainly relate to doctoral students, but there were also instances of taught students being framed in this way:

‘the Masters courses … are geared towards developing new data scientists … who are not necessarily aimed at going on to the PhD level, but to create impact’ (P29)

‘when you have some years from researching something, teaching that, and then your students go to the industry and then they come back… I think there is a circle there between teaching and research … and it completes the circle.’ (P39, STEMM, UK)

Participants gave examples of their doctoral students playing key roles in realising the impact of their broader programmes of research. In some cases, doctoral students led on the ‘impact’ strand of wider projects. For example, one participant explained how some of their PhD students spend around half a year ‘working on more of the theory or analytical side, from the Institute’ and spend the rest of the time ‘doing extended internships, working on applying the ideas in practice… partly funded by these industry partners’ (P10, STEMM, USA).

Another participant explained how their PhD students worked on applying the laboratory’s technology to meet the NSF ‘broader impact’ criteria of grants. This participant emphasised the value added of having sections of grants focused on impact:

‘with bigger grants, you have enough people support, they could easily each dedicate 10% of their time, all of a sudden you’ve got enough human power, it really adds up to something.’ (P71, STEMM, USA)

Some reflected that students can be highly motivated by the idea of impactful research:

‘*they* [students] were motivated by having an impact. …They were struggling with the notion that people weren’t already doing this [trying to apply their research])’. (P5, STEMM, UK)

Another participant agreed that ‘students want to do impactful research’ (P31, STEMM, UK) and suggested that this may be because students, unlike academics, do not yet feel pressured to meet generic academic metrics and therefore can be more focused on their genuine societal values.

For our STEMM participants, impact is integrated into the education function via academics regularly looking for strategic opportunities to combine the two missions into simultaneous activities. In particular, doctoral students work on applications of the broader research programme, either as part of their main research project or broader training. This is typically organised strategically so that it directly benefits external partners/users or fulfils formal impact requirements.

#### Computer scientists’ integration of the impact mission and education function

Generally, computer scientists spoke more deeply about their educational role as part of their academic identity. Several framed this in terms of contributing to their field by developing the next generation of experts. For them, education itself is a form of impact, potentially even the main form of impact that they are likely to have, in the sense that they are advancing the future of their discipline, which in turn will impact wider society:

‘my impact is going to be not proportional to what I can generate, but what my group generates. A lot of my attention right now is around that, mentoring PhD students… I suppose I’m evaluating my success in terms of like, output of my students’ (P50, CS, USA)

‘I probably take on more [PhD students] than most academics do generally … it’s one way I measure impacts. … teaching students is something I see is really impactful.’ (P63, CS, UK)

‘My goal is to try to make strides on this [aim of the field] right now. And to do that, you know, I would say, there’s the fundamental research, but most importantly, there's mentoring of students.’ (P46, CS, USA)

Computer scientists attached significant meaning to their educational role in our interviews. Several explained that the opportunity to work with and train students is the reason they chose to work in academia:

‘that’s kind of what keeps me in the academia game a little bit, the opportunity you have for good to create an environment for learning and knowledge to bring in students. …I want to see teaching as core to what we do… It's about kind of bringing through the next generation of researchers.’ (P63)

‘I generally think that one of the best ways to make the world a better place is to work for educational purposes, and universities are a good place that is less profit oriented than companies’ (P34)

‘working in industry didn’t appeal compared to the university. The advantage of the university is basically building up these things with the smart young people’ (P73, CS, Australia))

Interestingly, several computer scientists explained that their motivation to develop students even informs their research topics and approaches:

‘I plan to start writing a new proposal, and that’s really for supporting my students, whatever kind of ideas I’m going to put into that proposal, they are going to be more aligned with topics that are popular … for my students.’ (P60)

‘I value the research projects that I do as a way of training my students’ (P57, CS, USA)

‘I think a lot about ... finding interesting problems that we can contribute to as part of the student’s training. …So I would say the training of not just PhD students, but undergraduates who work in the lab, master’s students who work in the lab, that's a really important output’ (P14)

Thus, computer scientists see themselves as integrating impact and education in the sense that by training the next generation of researchers, they are contributing to the long-term advances and impacts that the discipline will achieve.

## Discussion and conclusions

### Theoretical implications: the complex and contingent relationship between system-level developments and individual-level values

As Bentley et al. (2015) note, the mode 2 thesis is widely influential in higher education and research policy. It is widely accepted despite its breadth and scope, its resistance to empirical confirmation (Bresnen & Burrell, 2013) and available alternative theories of greater relevance to some national systems (Trippl et al., 2015). The thesis suggests a decreasing role for disciplines as a source of identity and community. In our study, STEMM participants were indeed emphatic and consistent in their drive for impact, exhibiting a close integration of impact with research and weak attachment to disciplinary goals. In Nowotny et al.’s terms (2001), the research was relatively ‘strongly contextualised’; while, by contrast, our computer scientist participants emphasised being motivated by intellectual values, with their disciplinary community remaining the key audience, and their research relatively ‘weakly contextualised’, which is not incompatible with the research being funded by organisations with an interest in impact (pp. 121, 131). For computer scientists, Henkel’s (2005) findings ring true: their disciplinary community sets the stakes underpinning their academic identity, and shifts in research policy have not greatly disrupted academic values or academic identities. This includes being highly motivated by their educational role, both as a value in its own right and as a means of contributing to their discipline.

The mode 2 theorists have always maintained that it is ‘emerging alongside’ mode 1, supplementing rather than supplanting it (Gibbons et al., 1994, p. 11). In this sense, our study reinforces the complexity of research, involving non-uniform modes of conduct and societal relations. However, one of the first claims about mode 2 research is that it occurs ‘most frequently in those areas which currently define the frontier’ (Gibbons et al., 1994, p. 1). It is thus striking that many of our participants seem far closer to mode 1 than mode 2 in their practices and values, given their focus on AI—arguably the field which currently defines the frontier more so than any other. From our perspective, the key theoretical implication of our study—from which we also derive policy implications below—is that research and its relation to society are too complex to necessarily expect neat consistency between the various components and processes. On the one hand, our study shows that the impact agenda is deepening the integration of (academic) knowledge production with contexts of practice and application, thereby reinforcing (as well as resulting from) the tendencies towards mode 2. On the other, we find that this can play out in vastly different ways even amongst a relatively homogenous group of ‘frontier’ researchers, namely, STEMM academics with an AI specialism, including more traditional mode 1 practices and values. Analysing these issues through the lens of researcher identities and values, we find a multiplicity of sources of identification, community and purpose: the knowledge and technological needs of (particular groups within) society and industry; the expectations and demands of funders and academic institutions; the educational role of academics; and the internal dynamics and interests of a disciplinary community. In their survey of academics’ research activity and goals across fifteen countries and five continents, Bentley et al. (2015) conclude that mode 2 must still be seen as ‘emergent’ (p. 690) and that shifts at the level of individuals may not align directly with shifts at the system level. Our findings reinforce that any relationship between system-level tendencies and individual level values and motivations are contingent upon a complex range of factors.

### Policy implications: the need for understanding of how impact relates to research and education

From a policy perspective, it may be tempting to conceptualise AI as an exemplar of the linear research-impact model, whereby academics with different disciplinary backgrounds specialise at different stages of AI creation, development, testing, application, implementation, commercialisation, etc. The present paper does not speak to the veracity of such a model but does indicate that such a model, regardless of its usefulness, may give the misleading impression that that this is how the academics themselves understand their work. Our study suggests that it this is not necessarily the case. Despite academic and policy ideals to break down or transcend disciplinary boundaries, it remains important to acknowledge that disciplines are crucial sites of motivation, identity and community. As such, they remain an important reality for policy to contend with. Indeed, it may even be counterproductive to attempt to undermine disciplinarity, at least in some cases. For example, for our computer scientists, orientation to impact is closely related to disciplinary identity, collapsing a presumed disciplinary/impact dichotomy. Our study suggests that the two orientations may at times be mutually compatible and supportive.

Additionally, our findings suggest that policy should take more interest in how research, impact and education work together. From our STEMM participants, we learned that impact is shaping how academics approach their educational role, and that students and graduates can be important mechanisms through which research-based impact is achieved. And from our computer scientists, we learned that for some academics, their educational role can be a key or even primary way in which they see themselves as having an impact on society. The relationship between research, education and impact missions interrelate is complex, potentially mutually reinforcing and insufficiently understood. One immediate policy implication of this is that more needs to be done to understand unintended effects of policies or practices dividing research and teaching functions in universities.

Bentley et al. (2015) conclude with ‘a warning against policies striving for clear division in the higher education landscape between institutions primarily doing basic research and others applied’ (p. 705). Our study similarly suggests a warning against policies that divide research and education or that assume a dichotomy between disciplinary orientation and impact orientation.

#### Limitations

Qualitative methods inevitably have limitations around generalisability. Our decision to recruit only those with an AI focus means we cannot say how different our findings would be if we had sampled the same disciplinary categories (computer science and STEMM academics) without the thematic focus, or based on some other shared focus. However, this methodological decision also has strengths as it offers one way of increasing the specificity of our findings. For example, it would not be obvious in advance that computer scientists would diverge so significantly from other STEMM academics with a shared AI focus.

The paper is also limited in its empirical focus. Although Australia, the UK and the USA represent quite different contexts and systems, they also have much in common culturally and socio-politically. While the theoretical debates that this paper contributes to are genuinely ‘global’ (albeit disproportionately focused on high-income countries), the results might be different if they focused on a broader range of national contexts.

## Footnotes

1 A similar international survey found that the UK was about average on such issues (Bentley et al., 2015).

2 We identified universities with internal multi-departmental AI networks by first searching the term ‘artificial intelligence’ in the database of each national funding agency (ARC, NSF, UKRI) and creating a list of those universities that featured most frequently in the results. We then searched the phrase ‘artificial intelligence’ in all these universities’ homepage search bars. Only a small number of universities searched were found to have instituted a visible cross-institutional network. In each country we selected the university that seemed to provide the best opportunities for our recruitment purposes by offering a large list of potential participants from a broad range of disciplines.
